# Severe childhood lichen planus pemphigoides after hepatitis A vaccination

**DOI:** 10.1002/ski2.94

**Published:** 2022-01-19

**Authors:** M. Lahouel, A. Aounallah, S. Mokni, B. Sriha, C. Belajouza, M. Denguezli

**Affiliations:** ^1^ Department of Dermatology Farhat Hached Hospital of Sousse Sousse Tunisia; ^2^ Department of Pathology Farhat Hached Hospital of Sousse Sousse Tunisia

## Abstract

**Background:**

Lichen planus (LP) pemphigoides (LPP) is a very rare autoimmune bullous disorder, that is, exceptional in children.

**Case Report:**

We report a case of LP pemphigoides with severe cutaneous and mucosal involvement in an 8‐year‐old girl who consulted for multiple vesicular and bullous lesions associated with shiny erythematous‐purple plaques. The eruption occurred 2 months after vaccination against hepatitis A virus. The diagnosis of LP pemphigoides was confirmed by histopathology and immunofluorescence examination. The patient received oral corticosteroid therapy with rapid improvement.

**Conclusion:**

To our knowledge, this is the first report of LPP following hepatitis A vaccination, among adults and children.

## Graphical abstract

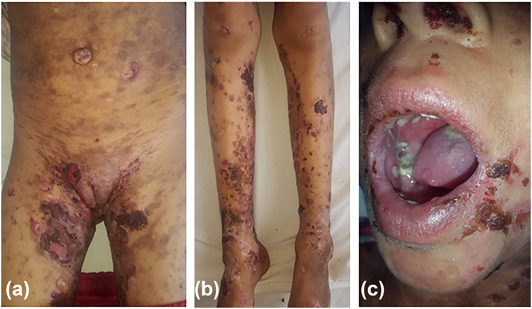

## 1 INTRODUCTION

Lichen planus (LP) pemphigoides (LPP) is a rare, autoimmune, subepidermal bullous disease of unknown aetiology and pathogenesis.^1^ Clinical, histopathological and direct immunofluorescence evaluation is important for the diagnosis of this entity. Its occurrence in paediatric patients is exceptional. Most of cases are idiopathic; however, anecdotal reports of LP pemphigoides induced by drugs and infections have been also described.^1^ To our knowledge, this is the first report of severe childhood LPP following hepatitis A vaccination.

## 2 REPORT

**FIGURE 1:**
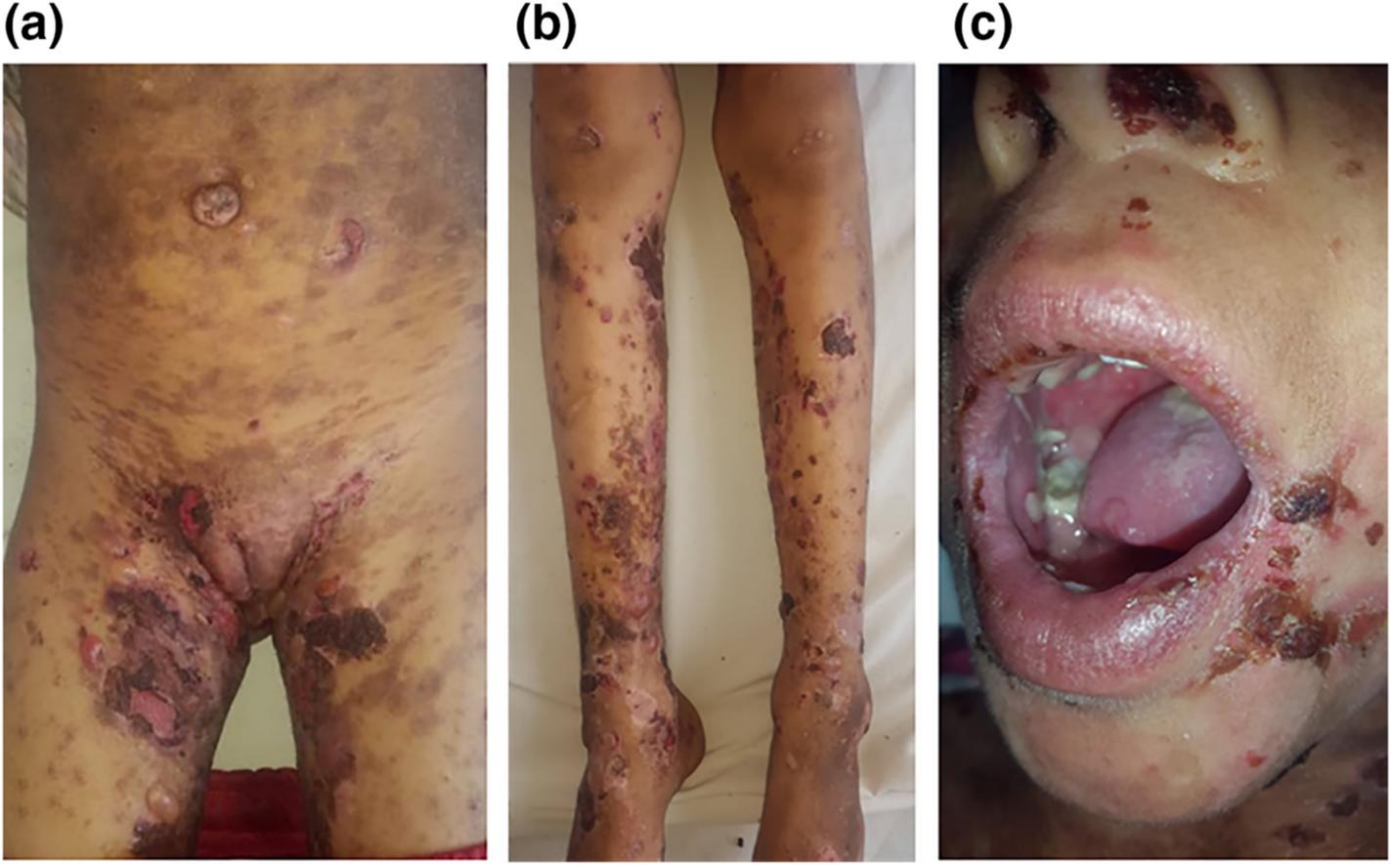
(a) Diffuse erythematous‐purple plaques of the trunk and genital area with blisters and crusted post‐bullous erosions (b) erythematous‐crusted patches of the limbs (c) perioral, intraoral and nasal crusted erosive lesions

**FIGURE 2:**
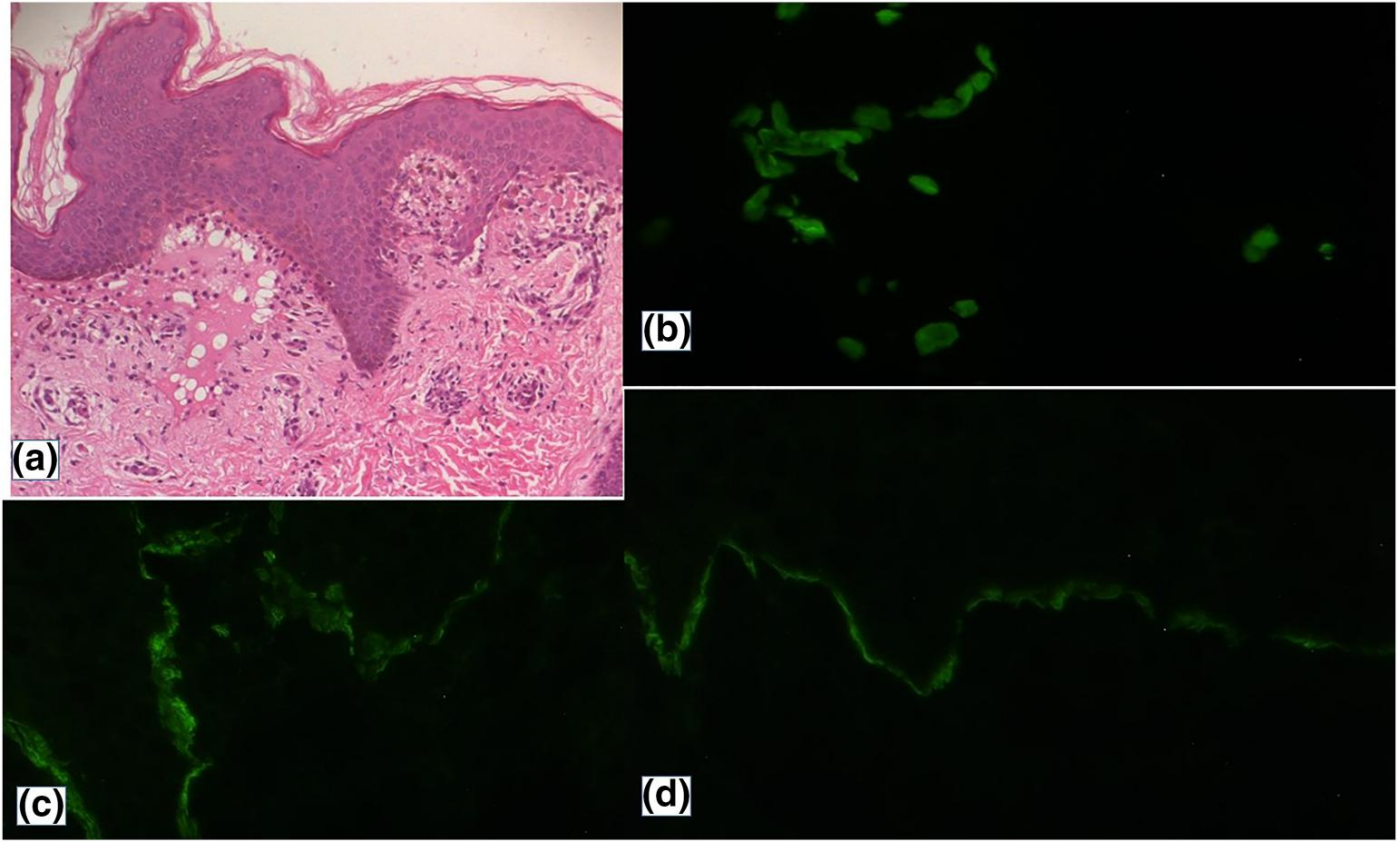
Histological features from a skin biopsy (a) (HE*40) biopsy of a bubble showing a sub epidermal blister surmounted by a regular and non‐necrotic epidermis containing neutrophils and eosinophils associated with a rich inflammatory infiltrate predominant in the superficial dermis, nibbling the basal layers associated with apoptotic keratinocytes (b–d) direct immunofluorescence from per bullous skin (b) IgM hyaline balls along the dermal‐epidermal junction (colloid bodies) (c) linear deposition of IgG along the dermal‐epidermal junction (d) linear deposition of C 3 along the dermal‐epidermal junction

An 8‐year‐old girl, with a medical history of hepatitis A at the age of 5 years, was admitted to our department with 2 months history of pruritic erythematous‐violaceous lesions on the abdomen that gradually spread to the whole body. Those lesions were followed 1 month later by the appearance of blisters and erosions. A family history of blistering or autoimmune diseases was denied, however, the patient reported having an inactivated, adsorbed hepatitis A vaccine (HAVRIX Infants and children 720 U/0.5 ml) 2 months before the eruption. Clinical examination showed multiple shiny violaceous papules and erythematous‐purple plaques localized on her hands and over her trunk and buttocks in large patches. These lesions were associated with tight clear vesicles and bubbles, localized on apparent normal skin as well as on lichenoid eruption. Erythematous eroded lesions were also seen on the trunk, abdomen and genital area (Figure 1a and b). Mucosal examination revealed lingual blisters with erosions on the buccal, nasal and vulvar mucosa (Figure 1c). The nails were not involved. Laboratory investigations, including complete blood count, chemical analyses and viral serology (hepatitis A, B and C) were normal. Histopathology of fresh bulla on lichenoid eruption was consistent with sub epidermal blister containing neutrophils and eosinophils in the bulla lumen and dense inflammatory cell infiltration in the papillary dermis (Figure 2a), nibbling the basal layers. The epidermis was regular with presence of apoptotic keratinocytes. Direct immunofluorescence examination of peri lesional skin showed globular staining with IgM (colloid bodies) at the derma‐epidermal junction (Figure 2b) and linear deposits of IgG and C3 at the basal membrane zone (BMZ) (Figure 2c and d). ELISA was performed and revealed antibodies directed against the 180 and 230 kDa polypeptides. The clinical, histopathological and immunofluorescence findings were compatible with the diagnosis of LP pemphigoides, possibly induced by vaccination against hepatitis A. The patient received prednisone (0.5 mg/kg/day) with a good response within the 10th day of treatment. Prednisone was tapered and discontinued over 10 months. During the 1‐year follow‐up, a mild itching with few papules occurred occasionally. The patient remains on intermittent topical steroids.

## 3 DISCUSSION

LPP was first described in 1892. Since then, Kaposi individualized two distinct forms of bullous eruptions of LP.^1^ The first form is bullous lichen planus, which is characterized by the formation of bubbles within the LP lesions themselves and are directly caused by inflammation of the dermis and degeneration of the basal layer. The second form is represented by LPP, which is a very rare autoimmune bullous dermatosis.^1^, ^2^ Clinically, LPP is characterized by the coexistence of typical LP lesions and strained blisters that are found both within LP lesions and at the level of the healthy skin, as the case of our patient. Histology of LPP reveals a sub epidermal blister. Direct immunofluorescence study of peri lesional skin shows linear deposition of IgG and/or C3 along the dermo‐epidermal junction associated occasionally to colloid bodies (IgM balls),^3^, ^4^, ^5^ which was compatible with our findings. Although LP pemphigoides was initially considered the simple coexistence of a LP and a bullous pemphigoid, the relationship between the two dermatoses appears to be more complex.^5^ LP pemphigoides has a younger mean age of onset than bullous pemphigoid, which appears most often after age 60. The evolving profile of LP pemphigoides is also less severe than bullous pemphigoid.^5^

The pathogenesis of LP pemphigoides remains incompletely elucidated.^3^ LP lesions might cause BMZ damage and expose BP180 antigens, which prompt the production of autoantibodies against BMZ, through a phenomenon termed ‘‘epitope spreading”. Circulating autoantibodies induce secondary sub epidermal bullous dermatosis.^2^, ^3^

In children, this condition is exceptional; indeed just 18 cases were reported.^2^, ^6^ This might be because of under‐reporting or misdiagnosis of other cases. The review of childhood LPP showed a predilection for males with a ratio of 3:1.^2^ The majority of the paediatric cases showed involvement of the palms and sole.^2^ A more diffuse involvement was found in our patient, more similar to the phenotype of adult LPP, which is characterized by more diffuse lesions and rarer palmar‐plantar involvement.^2^ A different antigen expression according to age could explain this difference suggesting that this antigen expression within LP lesions would lead to the formation of autoantibodies which do not only recognize sites limited to areas susceptible to developing LP lesions.

Lichen planus pemphigoides is most often idiopathic. However, cases of induced LPP by drug intake, namely angiotensin‐converting inhibitors, anti‐tuberculosis, PUVA, anti‐programmed death 1 (programed death 1) monoclonal antibody, were reported.^3^ LPP has been occasionally associated with internal malignancies, viral infections such as varicella,^6^ hepatitis B virus,^7^ chickenpox virus,^8^ pharyngitis,^9^ a body tattoo or a sun exposure^2^ have been reported. Association of LPP with viral infections, such as hepatitis B, was excluded due to the negativity of viral serology. Although the vaccines are generally safe, numerous reports highlighted the occurrence of autoimmune effects after single or combined multivaccine procedures. Only one paediatric erythrodermic LP pemphigoides following nonavalent human papillomavirus vaccination was recently described.^10^ In our patient, the vaccination against hepatitis A could be considered as a triggering factor for developing this autoimmune skin disorder. Molecular mimicry and bystander activation are possible mechanisms by which vaccines can cause autoimmune reactions. To our knowledge, this is the first report of LPP following hepatitis A vaccination among adults and children.

The course of LPP is variable. Indeed, several treatments have been proposed depending on severity of the clinical picture: topical and systemic corticosteroids, dapsone, methotrexate, azathioprine and tetracyclines.^2^, ^6^ The use of oral corticosteroids is controversial in children because of their side effects in this age group.^1^ Therefore, systemic steroids may be reserved as a second line therapy after dapsone, which is known to be effective and relatively safe, especially in children. The prescription of systemic corticosteroid therapy in our patient was decided based on the severity of the clinical presentation.

In conclusion, our case illustrates this rare and often misdignosed entity called LPP and emphasizes its potential severity. The triggering role of vaccination against hepatitis A deserves pharmacological exploration.

## CONFLICT OF INTEREST

None for all authors.

## AUTHOR CONTRIBUTIONS

**Maha Lahouel:** Conceptualization, Data curation, Formal analysis, Funding acquisition, Investigation, Methodology, Project administration, Resources, Supervision, Validation, Writing – original draft, Writing – review & editing; **Amina Aounallah:** Conceptualization, Funding acquisition, Project administration, Resources, Validation, Writing – review & editing; **Sana Mokni:** Data curation, Funding acquisition, Methodology, Software, Validation, Writing – original draft; **Badreddine Sriha:** Funding acquisition, Investigation, Resources; **Colandane Belajouza:** Conceptualization, Formal analysis, Investigation, Resources, Supervision, Visualization, Writing – review & editing; **Mohamed Denguezli:** Investigation, Methodology, Project administration, Visualization, Writing – original draft, Writing – review & editing.

## Data Availability

The data used to support the findings are included within the article.
